# Treatment of Pasteurella multocida Cervical Epidural Abscess

**DOI:** 10.7759/cureus.25507

**Published:** 2022-05-30

**Authors:** Mohamed Abdelraheem, Yousif Mohamed, Elaine Houlihan, Odhran Murray

**Affiliations:** 1 Orthopaedics and Traumatology, University Hospital Galway, Galway, IRL; 2 Trauma and Orthopaedics, Letterkenny University Hospital, Letterkenny, IRL; 3 Microbiology, Galway Univeristy Hospital, Galway, IRL; 4 Trauma and Orthopaedics, Galway University Hospital, Galway, IRL

**Keywords:** orthopaedics surgery, epidural abscess, cervical spine, pasteurella multocida, microbiology

## Abstract

A 51-year-old left-handed Caucasian female with no significant medical history presented with a two-week history of severe neck pain and bilateral upper limb weakness. Neurological examination revealed weakness and altered sensation in the C5-T1 distribution bilaterally, more severe on the left with Medical Research Council’s scale (MRC scale) of muscle power grade 3/5 and 4/5 on the right with upper motor neuron signs. Short-TI Inversion Recovery (STIR) and T2 weighted MRI imaging revealed increased signal at the C6-7 disc representing discitis, as well an anterior epidural collection from C5 to C7, with associated cord compression. The patient underwent an emergency anterior cervical corpectomy of C6, drainage of the epidural purulent collection, and insertion of a cage and plate. Some tissue and pus samples were sent to the microbiology laboratory for analysis, and the organism Pasteurella multocida was identified on all samples. The patient clinically and biochemically improved with operative management and a prolonged course of intravenous ceftriaxone. A peripherally inserted central catheter (PICC) line was placed and the patient was discharged on eight weeks of intravenous ceftriaxone and ongoing physical therapy.

## Introduction

Pasteurella multocida is an anaerobic gram-negative coccobacillus, which is the cause of a range of diseases in mammals and birds. It is also known to be responsible for zoonotic infections in humans [1], which typically is a result of pet bites or scratches. These bacteria colonize the normal oral, and upper respiratory tract [1-3], and gastrointestinal flora of many animals [4,5]. Diseases are primarily transmitted via animal bites, and scratches [6]. The most common disease manifestation of P. multocida includes local skin infection, and sometimes followed by local abscess formation, or lymphadenopathy [2-5,7,8]; however, it has also been known to cause osteomyelitis [7], sepsis [1,2,9], bacteremia [2-4,10], visceral infections [7], and other sites [3-5,7-12].

To our knowledge, there are several prior reported cases of P. multocida-induced spinal epidural abscess secondary to animal interaction [3]. Herein, we report a case of a previously well female with no documented history of an animal bite who developed a cervical epidural abscess secondary to P. multocida infection.

## Case presentation

A 51-year-old woman with no significant medical history presented to the Emergency Department with a 12-day history of intractable neck pain radiating to the left shoulder following a short prodromal period, including pharyngitis with no association with fever or tachycardia. She attended her GP and was prescribed antibiotics (oral Co-Amoxiclav 500/125mg eight hourly) and analgesia, including an IM injection during a home visit by her GP. She was then referred to a tertiary facility for an MRI cervical spine after which she was transferred for specialized orthopedic care in another tertiary hospital for assessment and further investigations. Her neck pain was constant, dull, and did not fit any dermatomal distribution. The patient reported a decline in fine motor skills and upper limb weakness, which progressed to include lower limb weakness and inability to stand in the days leading up to her admission. She was systemically well and afebrile on admission but reported feeling “hot and sweaty” at home. There was no objective weakness on the admission examination but objective night sweats were reported on the first night of admission. Her neurological examination revealed mild left deltoid (C5) Medical Research Council’s scale (MRC scale) of muscle power grade 4/5 weakness, left elbow extension (C7) 3/5, left wrist dorsiflexion (C6), finger abduction (T1), and long finger flexion (C8) 3/5 weakness with altered sensation over the aforementioned dermatomes. Examination of the contralateral side revealed 4/5 weakness over the same dermatomes with no altered sensation. The lower limb examination was unremarkable; however, her gait was ataxic. There was no muscle wasting or fasciculations observed. Of particular note, there were no breaks in the skin observed, i.e., wounds, and there was no skin rash reported.

**Figure 1:**
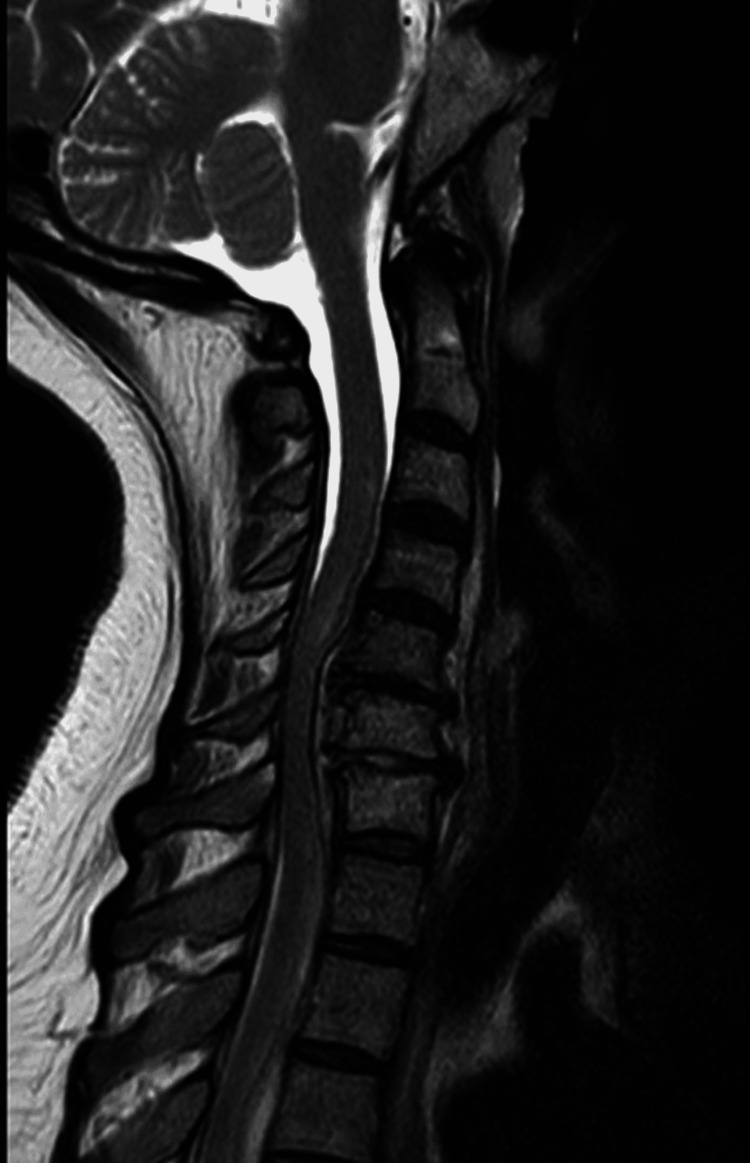
Sagittal T2 MRI showing a ventral C5-C6-C7 cervical epidural mass causing cord compression and displacement.

An MRI of the cervical spine showed an increased signal of C6-7 disc and adjacent endplates on T2 weighted and short-TI inversion recovery (STIR) sequences representing discitis, as well as an anterior epidural collection from C5 to C7, with associated cord compression (Figure 1). There was an altered cord signal from C4-C6 suggesting myelomalacia. Inflammatory markers were abnormal. Erythrocyte sedimentation rate (ESR) was 135 mm/hour (0-20 mm/hour), C-reactive protein was 202 g/dL (<0.5 mg/dL) and was 382 in the second presentation to a private hospital before transfer and her white cell count was elevated at 15 x 10^9^/L (4-10 x 10^9^/L). Blood cultures were taken on admission and were incubated for five days. The blood cultures remained sterile. Of note, one dose each of ceftriaxone and clindamycin was given before retrieval of blood cultures.

Based on the patient’s initial presentation to the Emergency Department, biochemical results suggesting infection, and MRI findings consistent with discitis with associated epidural abscess, the patient was commenced on intravenous antibiotics including ceftriaxone and clindamycin. She was transferred to the care of the orthopedic service for surgical intervention.

**Figure 2:**
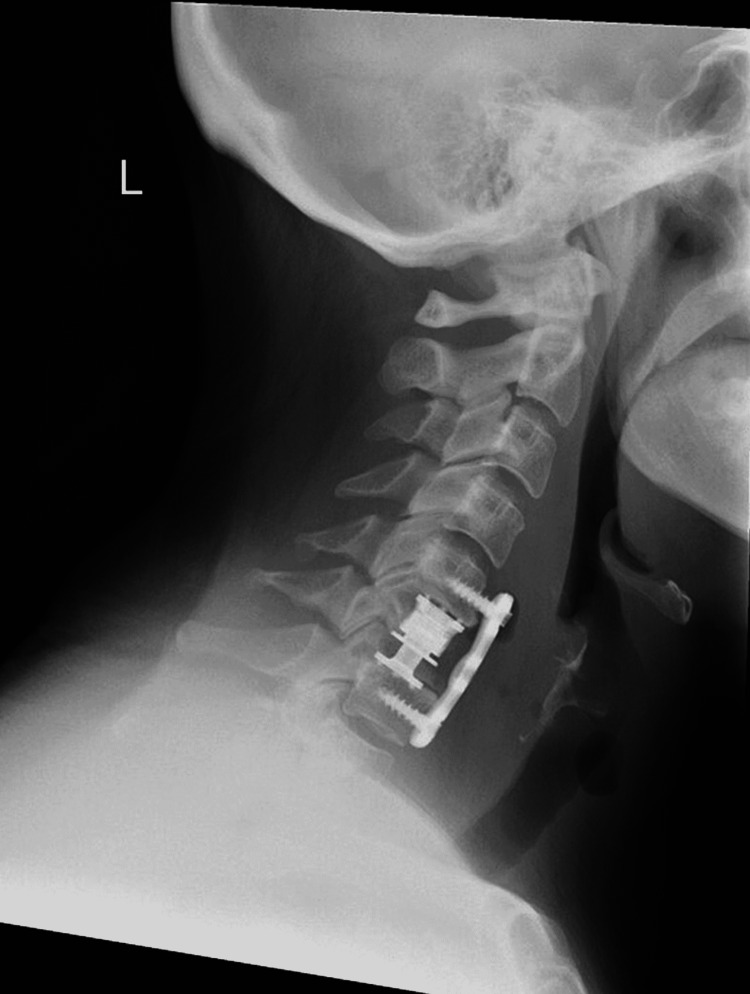
Lateral radiograph of the cervical spine demonstrating a C6 corpectomy, expandable cage, and plate fixed from C5 to C7, four weeks postoperatively.

She underwent an urgent anterior cervical corpectomy at C6, with debridement of the adjacent epidural abscess. Drainage of around 2-3mL of purulent material behind the bodies of C5 and C7 was achieved with a fine-tip blunt nerve root hook Thorough irrigation and end-plate perpetration were performed and the c-spine was stabilized with an expandable titanium cage and anterior cervical plate (Figure 2). Demineralized bone matrix allograft was packed into the cage and under the plate to encourage future fusion.

**Figure 3:**
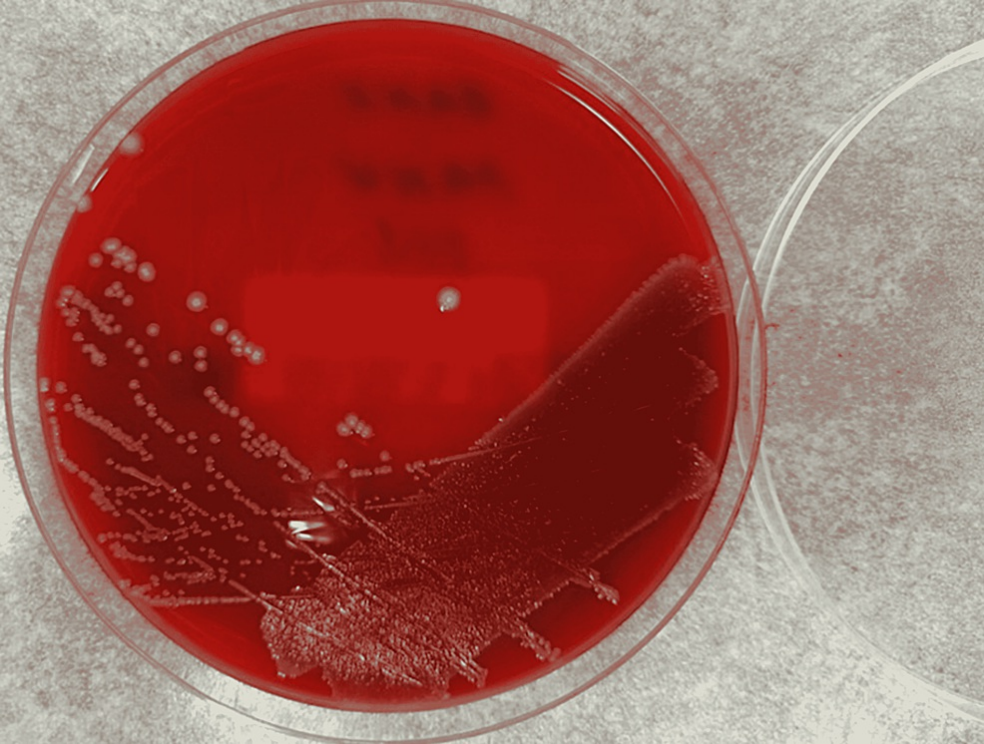
Blood agar plate showing non-hemolytic grey translucent colonies.

Multiple samples including tissue and pus samples were sent to the microbiology laboratories. Tissue from C6/C7 vertebra revealed a moderate white blood cell count at 5-20 white blood cells (WBC) per high power field, and no organisms were seen on the Gram stain. The samples were set up for culture, and growth was observed on the blood agar plate on day one of incubation. The colonies on the blood agar plate were grey and were convex, smooth, and translucent (Figure 3). The isolate was identified using the matrix-assisted laser desorption/ionization-time-of-flight (MALDI-TOF) as P. multocida. As advised by the Clinical Microbiologists, the patient was commenced on intravenous ceftriaxone 2g once daily. Susceptibility testing via disk diffusion was performed on the P. multocidaisolate confirming its sensitivity to penicillin, tetracyclines, and cotrimoxazole. Interpretation of susceptibility testing was performed using criteria provided by European Committee on Antimicrobial Susceptibility Testing (EUCAST).

In addition to IV antibiotics, the patients received three weeks of extensive inpatient physiotherapy and occupational therapy to aid clinical and functional recovery. For completion, a trans-thoracic echocardiogram was normal, and no valvular vegetations were observed.

## Discussion

P. multocida is a gram-negative coccobacillus that exists as normal flora in respiratory, nasal, and mouth flora of livestock, domestic animals such as dogs and cats, and poultry. Disease transmission primarily occurs via animal licks, bites, and scratches, and is most commonly associated with transmission from cats or dogs; however it was reported in this study [13] that 34 out of 79 cases were non bite transmission with P. multocida isolated as the causative organism which explains the mode of transmission in our case. There are many subspecies of Pasteurella, most commonly is P. multocida, but other subspecies include P. canis, P. dagmatis, and P. stomatis. P. multocida is most commonly associated with skin and soft tissue infections, but can also be causative in cases of bone and joint infections, respiratory tract infections, and much less commonly meningitis, endocarditis, and bacteremia. The carrier rate of P. multocida is 70%-90% in cats and 20%-50% in dogs. Cat bites have a higher risk of infection, because of higher colonization with P. multocida, but also because cats have small, sharp teeth causing deeper wounds. P. multocida infection has been discussed many times in the literature. One case review reported 44 patients with P. multocida infections [14], 43% (n=19) of which presented without a documented animal bite or scratch. Interestingly, patients who presented without an animal bite were more frequently hospitalized, had a longer length of stay, and were primarily patients with comorbidities/immunosuppression. Of the 44 patients discussed in this paper, eight patients required ICU admission for sepsis-related complications, seven of which had no animal bite reported [2]. Another case review reported 62 cases of P. multocida osteomyelitis, 50 of which were related to direct inoculation. Of the 62 cases reported, there were eight cases of vertebral osteomyelitis, six cervical, and two lumbar.

In this study [15] including a similar presentation of a 56-year-old female with a cervical epidural abscess that was treated surgically and administration of antibiotics, she similarly presented with neck pain, stiffness, and limb weakness, this was the only case out of 53 patients with a cervical epidural abscess that was found to be caused by P. multocida, which supports how rare it is. The paper also suggests early surgical intervention before the onset of limb paralysis in a patient with progressive limb weakness; however, nonsurgical treatment with antibiotics could be sufficient in patients with only neck pain and/or stiffness.

## Conclusions

In conclusion, P. multocida should be considered as one of the causes of acute neck pain/stiffness with or without the presence of neurology. Non-bite transmission of P. multocida is quite common and should be suspected cause of spinal epidural abscess in the presence of animal or pet exposure, via inhalation or ingestion. Acute neck pain with progressive neurological deficits is a surgical emergency, mandating rapid investigation and initiation of appropriate treatment. Lower motor neuron signs suggest compression of nerve roots, whilst upper motor neuron signs point to compression of the spinal cord. Thorough history and examination are required to diagnose spinal infection. In the absence of systemic inflammatory response syndrome or sepsis, try and obtain samples for culture and sensitivity before commencing antimicrobial therapy. A multidisciplinary team approach including the primary surgical specialty, clinical microbiology, and/or infectious disease, improves patient outcomes in such cases.

## Appendices

Patient statement

The 12 days preceding my attendance at a private hospital and subsequent transfer to the orthopedic team were exceptionally difficult. The initial period from the sore throat through to intense pain in the left neck/shoulder was three days. The pain was the most intense pain I have ever experienced. During this time, I was attended to three times by two GPs. After beginning to lose power in my left arm, the GP referred me to a regional hospital for treatment and an MRI scan, from where I was discharged after an x-ray with different pain relief. I cannot explain the physical and emotional trauma that I experienced during this period. 

When I attended the A&E department, there were blood tests taken immediately after initial questioning. My understanding is that these blood tests confirmed the decision to perform an MRI scan. (My GP had requested this when he referred me to the first hospital. The A&E Department said it is not in their protocol to refer people for MRI scans and I was subsequently discharged.) I was also given pain relief which was significantly more effective than any I had to that point. The doctor explained that I needed to be transferred to a spine orthopedic surgeon, I had a severe infection, and they would be expecting me. I was met by a team that examined, questioned, and re-questioned me. The doctors explained fully what was going to happen, why, how, and when. After the surgery, the post-operative care across all teams was incredible. I had physiotherapy to assist with regaining balance, walking, building strength in my arms and wrists, particularly in the left side which was most impacted. There was occupational therapy to regain motor skills and rebuild, as initially I could not write or lift a cup to my mouth. The Surgical and Infectious Disease teams were in regular contact. Care and communication from all teams, both in hospital and post-discharge has been excellent, which is incredible in these times of COVID-19.

My current state is that I have some minor balance issues on my left side and my left hand is still highly sensitized, particularly the index and middle fingers. I continue working on my physiotherapy and occupational therapy exercises as I have been told that nerve damage can continue to repair for up to 18 months post-injury. I need to do this to know that I have done everything on my part to do justice to the work and attention proffered to me by the teams in the hospital. I am deeply grateful to all.

My lesson in hindsight is to request for blood tests to be taken if there is no improvement in symptoms.
